# A novel parameter is better than the AHI to assess nocturnal hypoxaemia and excessive daytime sleepiness in obstructive sleep apnoea

**DOI:** 10.1038/s41598-021-84239-0

**Published:** 2021-02-25

**Authors:** Changxiu Ma, Ying Zhang, Jiuyu Liu, Gengyun Sun

**Affiliations:** 1 Department of Respiratory and Critical Care Medicine, the First Affiliated Hospital of Anhui Medical University, Hefei, 230032 China; 2 Department of Respiratory and Critical Care Medicine, the Second Affiliated Hospital of Anhui Medical University, Hefei, 230601 China

**Keywords:** Diseases, Medical research

## Abstract

To evaluate whether the percentage of total sleep time spent with apnoea and hypopnoea duration time (AHT%) is better than the apnoea-hypopnoea index (AHI) for the assessment of nocturnal hypoxaemia and excessive daytime sleepiness (EDS) in patients with obstructive sleep apnoea (OSA). Patients with suspected OSA were enrolled. Polysomnography, Epworth Sleepiness Scale, self-administered surveys and anthropometric measures were performed. The efficiency of AHT% and the AHI was evaluated for nocturnal hypoxaemia and EDS. A total of 160 eligible participants were analysed. The median AHT% in normal, mild, moderate and severe OSA patients was significantly different in the four-group patients with OSA. Spearman rank correlations analysis found that the associations were stronger between AHT% with percentage of total sleep time and O_2_ saturation of < 90% and minimum nocturnal oxygen saturation than these parameters with the AHI. AHT% had a greater area under the curve than the AHI for predicting EDS in patients with OSA. AHT% was significantly higher in the EDS group. We present a novel parameter, AHT%, to evaluate nocturnal hypoxaemia and EDS in OSA patients. AHT% partially compensates for the shortcomings of the AHI. AHT% is better than the AHI for assessing nocturnal hypoxaemia and EDS. AHT% reflects different clinical characteristics associated with OSA from a new perspective.

## Introduction

Obstructive sleep apnoea (OSA) is a disorder characterized by recurrent complete or partial collapse of the upper airway and has gradually received widespread attention since the 1970s. Untreated OSA can result in numerous clinical outcomes^1^, including excessive daytime sleepiness (EDS)^2,3^, impaired quality of life^4^, motor vehicle accidents^5^, incident hypertension^6,7^, myocardial infarction^8,9^, stroke^10^, heart failure^11^, diabetes^12^, and all-cause mortality^13,14^.

Apnoea-hypopnoea index (AHI) was the common index to diagnosis and classify the severity of OSA^15^. The AHI was firstly endorsed by research groups in the early 1980s^16,17^. The AHI is defined as the average number of apnoeas and hypopnoeas per hour of sleep. Although easy to calculate, it does not take into account other useful information, including associated nocturnal hypoxaemia, sleep fragmentation, duration of each expiratory event and the distribution of the events^18^. With the deepening of clinical research, researchers have gradually found a poor correlation between the AHI and related complications. For example, the AHI was not consistently associated with sleepiness, quality of life, or reaction time, both at baseline and as outcome measures in patients with mild-moderate OSA^4^. The AHI has only a weak correlation with different daytime sleepiness measures^19–22^, also cannot fully estimate the risk for cardiovascular disease (CVD)^6,23^. There were even conflicting results in relation to mortality^10,13^.

The utility of the AHI is a gross oversimplification of a complex disease phenomenon^24^, it can only obtain limited information from the rich polysomnography (PSG) data. In recent years, in order to fully reflect the clinical manifestations of OSA, more and more sleep experts have tried to solve this problem in different tools^25^. First of all, the most commonly used indicators are related to conventional oxygen saturation, such as minimum nocturnal oxygen saturation (Min SpO_2_), oxygen desaturation index (ODI), percentage of total sleep time with an O_2_ saturation of < 90% (CT90%). Previous studies have shown that these oxygen indicators may be more effective in predicting adverse outcomes and all-cause mortality of CVD^6,26,27^, as well as postoperative complications of upper airway surgery and weight loss surgery^28,29^. However, the limitation of these indicators is that other hypoxic diseases and central sleep-disordered breathing cannot be excluded. Secondly, some novel parameters have been proposed in recent years. Hypoxic burden was determined by measuring the respiratory event-associated area under the desaturation curve from pre-event baseline^30^. Unlike the AHI, hypoxic burden strongly predicted CVD mortality and all-cause mortality. Another research team^31,32^ presented four novel parameters (obstruction severity, desaturation severity, obstruction duration, and desaturation duration) were evaluated the severity of OSA. These four parameters could bring new insight to the individual estimation of OSA. Thirdly, there are other potential but less used indicators^25^, such as hypoxia load^33^, heart rate variability^34^ and integrated grading system^35^, etc. However, deficiencies still exist for these indicators because no one indicator could cover all the characteristics of OSA.

We are actively exploring a better indicator to reflect the severity of OSA in our clinical work. The duration of respiratory events has a significant effect on the outcomes of OSA, but the AHI does not take this important factor into account. The maximum and mean duration of apnoeas and hypopneas are easily measured from PSG data, although event duration is not an accepted diagnostic parameter in clinical practice guidelines or expert consensus. Inspired by the CT90% of calculating the total duration for oxygenation below 90% throughout the night, we considered whether we could measure the duration of respiratory events throughout the night to reflect the severity of OSA.

Therefore, we try our best to search a novel parameter, the percentage of total sleep time spent with apnoea and hypopnoea duration time (AHT%) in patients with OSA, which considers the total duration of all respiratory events. After searching literatures, we found that the concept of “obstruction duration” proposed by Finnish scholars in 2013 was similar with AHT%. However, this team and the others have not explored the correlation between this parameter and clinical symptoms in depth in recent years. We speculate that AHT% is similar with the AHI to assess nocturnal hypoxaemia and EDS in OSA patients. Thus, the purpose of this study was to explore whether AHT% is better than the AHI to evaluate nocturnal hypoxaemia and EDS in OSA patients.

## Methods

### Study population

A retrospective cross-sectional study was conducted. All 247 patients with suspected OSA were consecutively recruited from August 2018 to December 2019 in Respiratory Sleep Center of the Second Affiliated Hospital of Anhui Medical University. The exclusion criteria included that: Age was less than 18 years; Central sleep apnoea accounted for more than 5 per hour; Previously, ventilator pressure titration and previous continuous positive airway pressure (CPAP) treatment were performed; Total sleep time was less than 5 h; Patients who had incomplete information. Each patient filled out a self-administered survey that included morbidity assessment for dozens of diseases, patients with a history of severe heart, liver, lung, neuromuscular diseases and renal insufficiency were not included in the study. This study was approved by the medical ethics office of the Second Affiliated Hospital of Anhui Medical University, and all patients provided written informed consent.

### Baseline data

Each participator completed a baseline examination. Sex, age and medical history (such as type 2 diabetes mellitus and hypertension) were obtained. Anthropometric measures were conducted along with a single measurement of resting blood pressure during the night of the sleep study. Anthropometric measures, such as weight, height, body mass index (BMI) and neck circumference (NC), were collected.

### Questionnaires

Patients were asked to complete the Epworth Sleepiness Scale (ESS) before PSG. The ESS is an effective screening instrument consisting of 8 questions assessing sleepiness tendencies while engaged in activities of daily living, with each item rated from 0 (not at all likely to fall asleep) to 3 (very likely to fall asleep). The total score ranges from 0 to 24, and subjectively quantified EDS was defined as a score ≥ 11^36^.

### Sleep monitoring

All participants underwent fully attended overnight PSG. PSG was performed using a polygraph system (Embla S4500, USA). Electroencephalography (C3-A2, C4-A1, O1-A2, O2-A1), bilateral electrooculogram, and electromyogram were performed. Airflow was monitored by a nasal pressure transducer and/or oronasal thermal sensor. Polysomnograms were scored by an experienced PSG technologist. All recordings were manually reanalysed based on the standard method by the American Academy of Sleep Medicine (AASM) in 2007. Apnoea was defined as a ≥ 90% decrease in airflow for at least 10 s, and hypopnoea was defined as a ≥ 30% reduction in airflow for at least 10 s accompanied by oxygen desaturation ≥ 4%. The AHI was categorized using commonly used clinical cutoff points: AHI < 5 (normal), 5 ≤ AHI < 15 (mild OSA), 15 ≤ AHI < 30 (moderate OSA), and ≥ 30 events/h (severe OSA). Total sleep time was computed based on manual sleep staging. The conventional diagnostic parameters are the AHI, Min SpO_2_ and CT90%. The percentage of total sleep time (min) spent with apnoea and hypopnoea duration time (min) is denoted as AHT%.

$${\text{AHT}}\% = \left[ {\frac{{{\text{total}}\;{\text{ apnoea}}\;{\text{ duration}}\;{\text{ time}} + {\text{total}}\;{\text{ hypopnea}}\;{\text{ duration}}\;{\text{ time}}}}{{{\text{total }}\;{\text{sleep }}\;{\text{time}}}}} \right] \times 100\%$$

### Statistical analysis

SPSS (version 21.0, USA) was used to establish a database and conduct statistical analysis. Shapiro–Wilk method was used to analyse quantitative data in normal distribution. Normally distributed quantitative data are described as the mean ± standard deviation (SD). Two independent samples t-tests were used for comparisons between two groups, and one-way ANOVA was used for comparisons between multiple groups. If the difference was statistically significant, pairwise comparisons between multiple groups were performed using Student–Newman–Keuls. Quantitative data with skewed distributions were described by medians (P_25_, P_75_), Mann–Whitney *U* tests were used for comparisons between two groups, and Kruskal Wallis tests were used for comparisons between multiple groups. Spearman correlation analysis was used to determine the correlations between data with skewed distributions. The qualitative data are described as frequencies and percentages, and Pearson χ^2^ tests were used for comparisons between groups. The area under the curve (AUC) was predicted based on a receiver operating characteristic (ROC) curve. *P* < 0.05 was considered the limit for statistical significance. PASS 15.0 statistical software was used to analyze power calculation in One-Way Analysis of Variance F-tests module.

### Ethics declarations

All procedures performed in studies involving human participants were in accordance with the ethical standards of the institutional and/or national research committee and with the 1964 Helsinki declaration and its later amendments or comparable ethical standards. This study was approved by the Second Affiliated Hospital of Anhui Medical University.

## Results

### Baseline characteristics of the sleep study cohort according to the AHI

Data were available for 247 participants, providing an analytical sample size of 160. Demographic characteristics and clinical information were shown in Table 1. AHT% was taken as the outcome variable to calculate the sample size. The results of power calculation showed that when α = 0.05 and β = 0.10, the minimum sample size required for each group was 4 people, so the sample size of this study met the needs of statistical analysis. Participants had an average age of 40.44 years at baseline (SD, 15.08) and 69.4% were male. The average BMI was 32.91 kg/m^2^ (SD, 9.46) and average NC was 42.59 cm (SD, 5.09). 36.9% of the sample had prevalent hypertension, and 11.3% had prevalent diabetes. Patients were classified into four groups based on the severity of OSA. The results showed that significant differences in age, NC, systolic blood pressure (SBP), diastolic blood pressure (DBP), ESS, AHI, AHT%, Min SpO_2_ and CT90% were found in the four groups. No difference of BMI was observed among four groups. The pairwise comparison between severe OSA and normal, mild and moderate OSA was statistically significant in terms of NC, SBP, AHI, AHT%, Min SpO_2_ and CT90%. The median AHT% in normal, mild, moderate and severe OSA groups was 0.7%, 4.85%, 12.68%, and 46.54%, respectively, and the differences were significant in the four-group comparison and in the pairwise comparison. AHI severity was higher in men and those with a history of hypertension, while the difference was not statistically significant in regard to the presence or absence of diabetes. Blood oxygen saturation was gradually decreased and blood pressure was gradually increased in parallel with the severity of OSA. There was no change of ESS and BMI among different groups.

**Table 1:** Baseline characteristics of the sleep study cohort according to the AHI.

	Whole population	Normal (n = 13)	Mild OSA (n = 30)	Moderate OSA (n = 19)	Severe OSA (n = 98)	χ^2^/F	*P* value
**Sex, n (%)**						12.264	0.007
Men	111 (69.4)	5 (38.5)	19 (63.3)	10 (52.6)	77 (78.6)		
Women	49 (30.6)	8 (61.5)	11 (36.7)	9 (47.4)	21 (21.4)		
Age, year	40.44 $\pm$ 15.08	25.92 $\pm 9.95$	38.40 $\pm$ 13.96^a^	41.21 $\pm$ 15.95^a^	$42.85\pm$ 14.81^a^	5.476	0.001
BMI, kg/m^2^	32.91 $\pm$ 9.46	32.00 $\pm$ 8.47	30.91 $\pm$ 9.17	32.19 $\pm$ 9.17	33.78 $\pm$ 9.40	0.795	0.499
NC, cm	42.59 $\pm$ 5.09	38.38 $\pm$ 3.01	41.12 $\pm$ 4.86	40.68 $\pm$ 3.66	43.97 $\pm$ 5.10^abc^	8.061	< 0.001
SBP, mmHg	128.30 $\pm$ 18.85	114.46 $\pm$ 16.64	124.03 $\pm$ 16.60	123.32 $\pm$ 17.50	132.41 $\pm$ 18.87^abc^	5.227	0.002
DBP, mmHg	81.86 $\pm$ 13.86	71.38 $\pm$ 11.52	78.40 $\pm$ 12.48	78.79 $\pm$ 9.42	84.90 $\pm$ 14.36^ab^	5.388	0.001
**Hypertension, n (%)**						15.983	< 0.001
No	101 (63.1)	12 (92.3)	24 (80.0)	14 (73.7)	51 (52.0)		
Yes	59 (36.9)	1 (7.7)	6 (20.0)	5 (26.3)	47 (48.0)		
**Diabetes, n (%)**						^- d^	0.476
No	142 (88.7)	13 (100.0)	26 (93.3)	14 (84.2)	84 (86.7)		
Yes	18 (11.3)	0 (0)	2 (6.7)	3 (15.8)	13 (13.3)		
ESS	8.00 (4.00,11.00)	6.00 (1.50.8.00)	6.00 (2.75,9.25)	9.00 (7.00,12.00)^a^	8.00 (4.00,12.00)	8.179	0.042
AHI, /h	50.45 (13.38,70.73)	1.50 (0.75,3.10)	9.25 (6.65,11.35)^a^	22.20 (19.00,27.00)^ab^	67.00 (53.78,88.18)^abc^	121.072	< 0.001
AHT%	30.04 (7.91,50.23)	0.70 (0.30,1.23)	4.85 (3.04,5.73)^a^	12.68 (10.65,13.77)^ab^	46.54 (32.99,58.69)^abc^	120.847	< 0.001
Min SpO_2_, %	91.85 $\pm$ 4.97	96.26 $\pm$ 0.65	95.26 $\pm$ 1.08	94.61 $\pm$ 1.39	89.69 $\pm$ 5.23^abc^	23.011	0.001
CT90%	12.78 (0.94,35.59)	0.0 (0.0,0.05)	0.36 (0.08,1.46)^a^	2.74 (0.65,4.82)^ab^	29.58 (14.80,52.32)^abc^	107.318	< 0.001

*AHI* apnoea-hypopnoea index; *OSA* obstructive sleep apnoea; *BMI* body mass index; *NC* neck circumference; *SBP* systolic blood pressure; *DBP* diastolic blood pressure; *ESS* Epworth Sleepiness Scale; *AHT%* the percentage of total sleep time spent with apnoea and hypoponea duration time; *Min SpO*_*2*_ minimum nocturnal oxygen saturation; *CT90%* percentage of total sleep time with an oxygen saturation of < 90%.

^a,b,c^The differences were statistically significant compared with the normal, mild and moderate AHI groups, respectively (*P* < 0.05).

^d^Fisher’s exact probability was used.

#### Correlation between AHT%, AHI and CT90% and Min SpO_2_ in OSA patients

**Figure 1:**
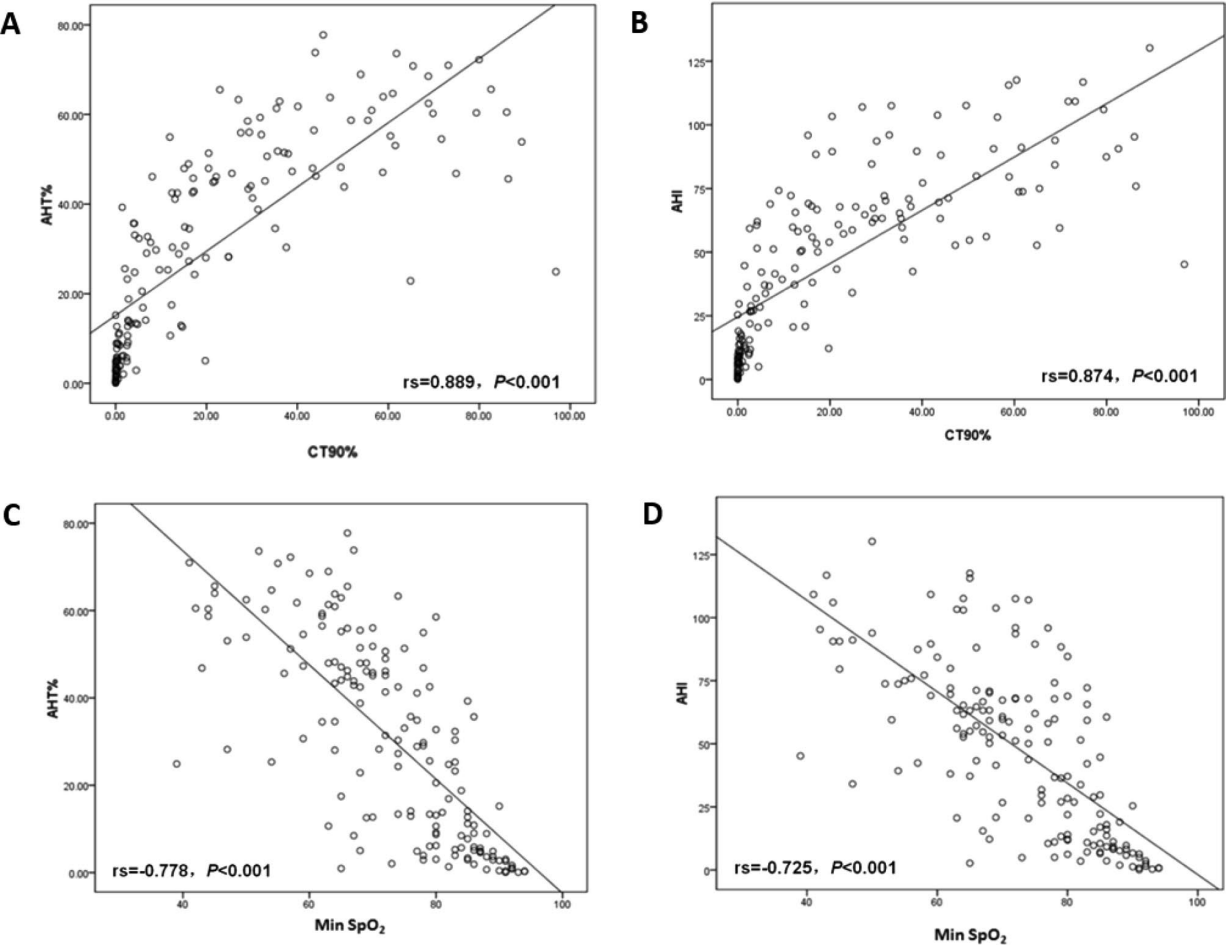
Correlation between AHT% and CT90% (**A**) and Min SpO_2_ (**C**), correlation between AHI and CT90% (**B**) and Min SpO_2_ (**D**).

 AHT% and AHI were regarded as independent variables, CT90% and Min SpO_2_ were regarded as dependent variables, respectively. The associations among AHT%, AHI, CT90% and Min SpO_2_ were analyzed using Spearman rank correlations. As shown in Fig. 1, AHT% (A, rs = 0.889, P < 0.001) and AHI (B, rs = 0.874, P < 0.001) were positively correlated with CT90%. Not only that, the associations between Min SpO_2_ with AHT% and AHI were explored. The results indicated that Min SpO_2_ was inversely associated with AHT% (C, rs = − 0.778, *P* < 0.001) and AHI (D, rs = − 0.725, *P* < 0.001). Moreover, the correlation between AHT% and CT90% and Min SpO_2_ was better than the AHI.

#### Comparison of ROC curves of AHT% and AHI for predicting ESS ≥ 11

**Figure 2:**
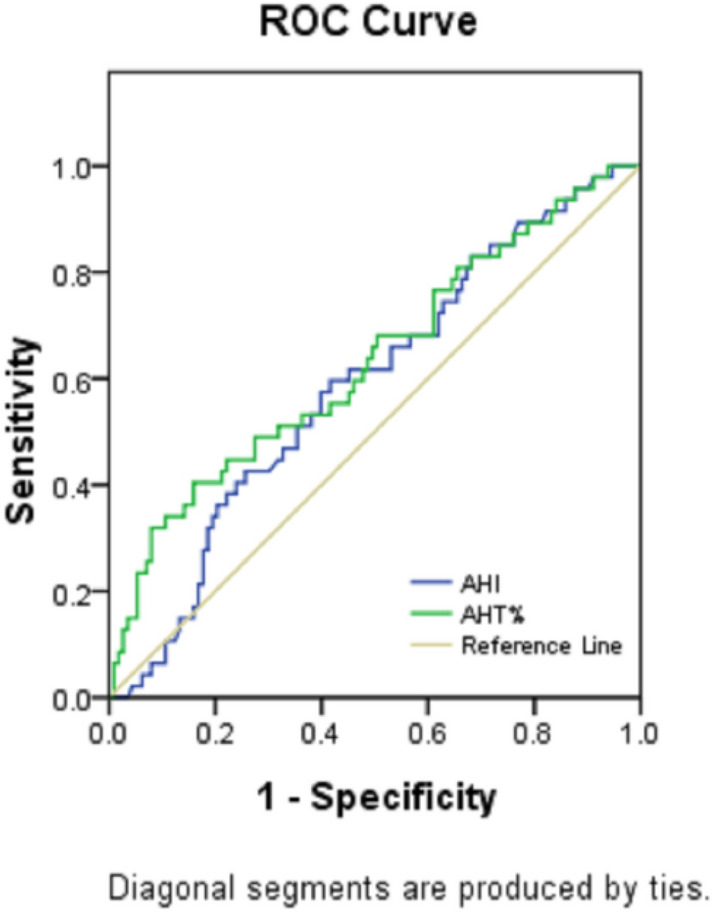
ROC curve analysis for AHT% and AHI for predicting ESS ≥ 11 in patients with OSA. The prediction performances of AHT% and AHI were assessed for clinically significant EDS (ESS ≥ 11). AHT% demonstrated a higher predictive accuracy than AHI for ESS ≥ 11.

Figure 2 and Table 2 show that AHT% (AUC = 0.632, *P* = 0.008) had a larger AUC than AHI (AUC = 0.588, *P* = 0.081) for predicting ESS ≥ 11 in patients with OSA, and the result was statistically significant (*P* < 0.05). As seen from the ROC curve, AHT% was better than the AHI for predicting EDS (ESS ≥ 11).

**Table 2:** Comparison of ROC curves of AHT% and AHI for predicting ESS ≥ 11.

Test result variable(s)	Area	Std. error^a^	Asymptotic Sig.^b^	Asymptotic 95% confidence interval	
				Lower bound	Upper bound
AHT%	0.632	0.050	0.008	0.535	0.730
AHI	0.588	0.048	0.081	0.493	0.682

The test result variable(s): AHI has at least one tie between the positive actual state group and the negative actual state group. Statistics may be biased.

Columns indicate metrics of regression performance, including the coefficient. Plots from the ROC analysis are shown in Fig. 2.

^a^Under the nonparametric assumption.

^b^Null hypothesis: true area = 0.5.

#### Characteristics of OSA patients classified by ESS < 11 or ESS ≥ 11

In the Table 3, we found that 47 patients (29.4%) were in the group of ESS ≥ 11, 34 (72.3%) of which were males. Age, AHT%, CT90% and Min SpO_2_ were higher in the ESS ≥ 11 patients than those in in the ESS < 11 group (*P* < 0.05). There was no difference of sex, BMI, AHI, NC, SBP, DBP, hypertension and diabetes between the two groups. In other words, AHT% significantly increased in the EDS group (ESS ≥ 11) but did not increase in the non-EDS group (*P* = 0.008). The AHI showed no significant difference between the two groups (*P* = 0.081). With age, OSA patients were more likely to develop EDS, regardless of the severity of the AHI, and there was no difference between men and women. Patients with EDS may have lower oxygen saturation but no increase in blood pressure.

**Table 3:** Characteristics of OSA patients classified by ESS < 11 or ESS ≥ 11.

	ESS		χ^2^/t/Z	*P* value
	< 11 (n = 113)	≥ 11 (n = 47)		
**Sex, n (%)**				
Men	77 (68.1)	34 (72.3)	0.275	0.600
Women	36 (31.9)	13 (27.7)		
Age, years	38.81 $\pm$ 14.72	44.36 $\pm$ 15.36	2.144	0.034
BMI, kg/m^2^	33.19 $\pm$ 10.46	32.21 $\pm$ 6.50	0.716	0.475
AHI, /h	43.30 (11.15,67.80)	59.50 (20.80,75.90)	1.746a	0.081
AHT%	25.56 (5.63,46.54)	43.36 (13.37,60.50)	2.632a	0.008
CT90%	7.72 (0.56,29.94)	24.83 (2.92,51.79)	2.666a	0.008
Min SpO_2_, %	74.75 $\pm$ 12.41	68.00 $\pm$ 13.12	3.083	0.002
NC, cm	42.37 $\pm$ 5.12	43.13 $\pm$ 5.02	0.856	0.393
SBP, mmHg	128.40 $\pm$ 18.46	128.06 $\pm$ 19.97	0.102	0.919
DBP, mmHg	81.45 $\pm$ 14.42	82. 83 $\pm$ 12.52	0.572	0.568
**Hypertension, n (%)**				
No	76 (67.3)	25 (53.2)	2.821	0.093
Yes	37 (32.7)	22 (46.8)		
**Diabetes, n (%)**				
No	101 (89.4)	41 (87.2)	0.153	0.696
Yes	12 (10.6)	6 (12.8)		

^a^Mann-Whitney *U* Test.

## Discussion

In the present study, we objectively evaluated AHT% and the AHI in 160 patients who had undergone full-night PSG and completed related questionnaires. The results showed that both AHT% and the AHI were closely related to CT90% and Min SpO_2_, while AHT% had a closer correlation than the AHI. AHT% was significantly different in EDS and non-EDS patients, but the AHI was not. AHT% had a stronger relationship with EDS than the AHI. Therefore, AHT% may be an alternative method for the assessment of nocturnal hypoxaemia and EDS in OSA patients.

The AHI, which is a traditional score used to determine the severity of OSA, is not a thorough or complete indicator to reflect the clinical manifestations and long-term prognosis of OSA. The application of the AHI has been widespread in OSA research for nearly 40 years, but as the gold standard for OSA diagnosis, its scientific and statistical properties have been questioned by some scholars. One of the limitations of the AHI may be that it does not take into account the total duration of respiratory events, while the duration is as important as the number of apnoeas and hypopnoeas in evaluating OSA severity. Respiratory event duration includes two concepts, one is the total duration of respiratory events throughout the night, and the other is the mean duration of each respiratory event, both of which have been discussed by scholars. The researches on the total duration has been done by Finnish researchers, who call it “obstruction duration” which was calculated in a manner similar to AHT%^31^. The results suggested that patients with a similar AHI may in fact suffer from sleep apnoea-hypopnoea syndrome of very different severity. In terms of the mean duration, the general view is that the longer the mean duration of respiratory events, the more serious the consequences. The research results of Zhan et al.^37^ found that patients with a long mean apnea–hypopnea duration had significantly worse blood oxygen levels and sleep parameters. Saraç et al*.*^38^ also revealed that the patients with OSA with long average duration were found to have more negative effects of sleep apnea than the patients with short average duration. However, another study overturned the conventional understanding^18^. The result showed that short duration of respiratory events predicted a significant hazard ratio for all-cause mortality. Individuals with shorter respiratory events might be predisposed to increased ventilatory instability and/or augmented autonomic nervous system responses. According to the aforementioned studies, we found that the clinical characteristics of respiratory events duration and the relationship with long-term outcomes were still unclear, and deserved further study. Therefore, we chose the total respiratory event duration as our study object.

Intermittent hypoxaemia is recognized as a potential major factor contributing to the pathogenesis of OSA comorbidities. Intermittent hypoxaemia promotes oxidative stress, increases sympathetic activation and promotes inflammation^39^. Therefore, ODI, Min SpO_2_ and CT90% are commonly used parameters to determine OSA severity and develop treatment plans based on the results^8,39–42^. Xie et al. found that Min SpO_2_ < 85% was an independent risk factor for major adverse cardiac events in patients after myocardial infarction^8^. The study of Kuklisova et al. showed that CT90% independently predicted CPAP failure after adjustments for covariates, but the AHI did not^40^. Previous studies have shown that the severity of nocturnal hypoxaemia may be similar to or more useful than the AHI in predicting the risk of associated conditions, such as cardiac dysfunction, endothelial impairment, hypertension, and atrial fibrillation^42^. Therefore, our study focused on the correlations between AHT% and the AHI and oxygen saturation, the results showed that Spearman rank correlations between AHT% and Min SpO_2_ and CT90% were significantly stronger than those of the AHI. Among the two types of oxygen saturation, the stronger correlation was observed for CT90%. Moreover, Min SpO_2_ and CT90% may be affected by a variety of hypoxic diseases, such as obesity hypoventilation, chronic obstructive pulmonary disease, asthma, heart failure, and pulmonary embolism. The advantage of AHT% is that it reflects the impact of only sleep apnoea and hypopnoea, avoiding the impact of some comorbidities of OSA.

Some previous studies have shown that since symptoms do not correlate well with OSA severity as defined by the AHI, a number of tools based on symptoms and physiology of OSA have emerged. EDS is a commonly reported non-AHI measure in the OSA literature^43,44^ and may contribute to an increase in cerebrovascular diseases, cardiovascular disease, motor vehicle accidents and occupational injuries^3,45,46^. A recent study showed that patients with EDS had higher rates of major adverse cardiac events and reinfarction than those without EDS in post-myocardial infarction patients^3^. The presence of EDS in individuals with moderate and severe OSA was associated with a higher risk of major adverse cardiac events than the risk among those without EDS and may be an independent prognostic factor. Furthermore, EDS is often utilized to evaluate whether CPAP and surgery therapy have curative effects and improve quality of life^44,47,48^. Therefore, our study evaluated the differences between AHT% and the AHI in EDS and non-EDS groups and assessed which one had closer correlation with EDS. The results were consistent with previous studies, the EDS group (ESS ≥ 11) had a significantly higher AHT%, but the AHI did not increase significantly. As shown in Table 1, the ESS did not increase significantly in patients with severe OSA compared to those with mild-to-moderate OSA. In short, AHT% is superior to the AHI for predicting EDS.

This article has several limitations. First, of the 160 patients who underwent PSG monitoring, 55 underwent sleeve gastrectomy. The incidence of OSA in this population is extremely high, and there may be selection bias. Second, long-term outcomes of OSA, such as cardiovascular risk and mortality, were not followed up. At present, these patients are being followed up regularly, and we will further explore the value of AHT% in long-term outcomes of OSA. Third, because the time span of this study is not too long and the number of cases is limited, the severity of AHT% is not graded to evaluate whether AHT% has more positive significance in guiding treatment.

Although the AHI is less than perfect, it will be difficult to discontinue the widespread application of the AHI in a short period of time because the AHI is too deeply rooted in the field. Every sleep-related parameter has its inherent disadvantages, and it is difficult to have a one-size-fits-all parameter that takes into account all aspects. Perhaps we can refer to the diagnostic criteria for severe pneumonia, which uses all the important factors as evaluation indicators and then evaluates disease severity according to the total score. Further clinical studies may be needed to explore how to integrate related indexes of respiratory events, hypoxaemia, the distribution of the events and other indicators.

We present a novel parameter, AHT%, to evaluate nocturnal hypoxaemia and EDS in OSA patients. AHT% takes into account the total duration of apnoeas and hypopnoeas in OSA patients, and partially compensates for the shortcomings of the AHI. The results reveal that AHT% is better than the AHI for assessing nocturnal hypoxaemia and EDS. This paper does not overrule the role of the AHI, but suggests that AHT% reflect different characteristics associated with OSA from a new perspective.

## Data Availability

All data included in this study are available upon request by contact with the corresponding author.
